# Epigenetic control of epilepsy target genes contributes to a cellular memory of epileptogenesis in cultured rat hippocampal neurons

**DOI:** 10.1186/s40478-017-0485-x

**Published:** 2017-10-31

**Authors:** K. Kiese, J. Jablonski, J. Hackenbracht, J. K. Wrosch, T. W. Groemer, J. Kornhuber, I. Blümcke, K. Kobow

**Affiliations:** 1 Institute of Neuropathology, Friedrich-Alexander-University Erlangen-Nürnberg, Universitätsklinikum Erlangen, Schwabachanlage 6, 91054 Erlangen, Germany; 2 Department of Psychiatry and Psychotherapy, Friedrich-Alexander-University Erlangen-Nürnberg, Universitätsklinikum Erlangen, 91054 Erlangen, Germany

**Keywords:** Primary neuronal cell culture, Hippocampus, Epigenetic, Cellular memory, Epilepsy

## Abstract

Hypersynchronous neuronal excitation manifests clinically as seizure (ictogenesis), and may recur spontaneously and repetitively after a variable latency period (epileptogenesis). Despite tremendous research efforts to describe molecular pathways and signatures of epileptogenesis, molecular pathomechanisms leading to chronic epilepsy remain to be clarified. We hypothesized that epigenetic modifications may form the basis for a cellular memory of epileptogenesis, and used a primary neuronal cell culture model of the rat hippocampus to study the translation of massive neuronal excitation into persisting changes of epigenetic signatures and pro-epileptogenic target gene expression. Increased spontaneous activation of cultured neurons was detected 3 and 7 days after stimulation with 10 μM glutamate when compared to sham-treated time-matched controls using calcium-imaging in vitro. Chromatin-immunoprecipitation experiments revealed short-term (3 h, 7 h, and 24 h) and long-term (3 d and 2 weeks) changes in histone modifications, which were directly linked to decreased expression of two selected epilepsy target genes, e.g. excitatory glutamate receptor genes *Gria2* and *Grin2a*. Increased promoter methylation observed 4 weeks after glutamate stimulation at respective genes suggested long-term repression of Gria2 and Grin2a genes. Inhibition of glutamatergic activation or blocking the propagation of action potentials in cultured neurons rescued altered gene expression and regulatory epigenetic modifications. Our data support the concept of a cellular memory of epileptogenesis and persisting epigenetic modifications of epilepsy target genes, which are able to turn normal into pro-epileptic neurons and circuits.

## Introduction

Temporal lobe epilepsy (TLE) is the most frequent focal epilepsy in humans and often associated with an initial precipitating injury, such as brain trauma, inflammation or prolonged febrile seizures, followed by a clinically silent latency period before onset of chronic recurrent seizures [1, 2]. Despite tremendous research efforts to describe molecular pathways and signatures of epileptogenesis, pathophysiological mechanisms leading to chronic epilepsy remain to be clarified. Gene expression profiling studies identified many genes to be differentially expressed in chronic TLE. Comprehensive gene pathway analysis suggested inflammation, stress, synaptic transmission and plasticity to play a role [3–7]. More recent data suggested epigenetic mechanisms, including DNA methylation, histone modifications, chromatin remodeling and non-coding RNAs as key upstream mechanisms deregulating gene expression, thereby promoting epileptogenesis [8–14]. These studies identified locus-specific as well as genome-wide alterations in DNA methylation patterns in different animal models or human surgical brain samples, compatible with a compromised gene regulation machinery underlying epileptogenesis. Furthermore, hyper- and hypoacetylation of histone H4 have been reported for several gene promoters following an initial precipitating injury, e.g. status epilepticus, and seem to be independent of the analyzed model [15–18]. Histone modifications are, therefore, of particular interest and capable to promote the epileptogenic process. Here we asked if neuronal hyperexcitation alters the epigenetic machinery of hippocampal neurons towards the previously described pro-epileptogenic cellular signature.

We intended to induce rhythmic hyperexcitation in cultured hippocampal neurons with 10 μM glutamate, to be documented by live-cell calcium imaging [19, 20]. Chromatin immunoprecipitation was performed at various time intervals to study epigenetic histone modifications, i.e. H4 acetylation as well as H3K4, H3K9 and H3K27 trimethylation. Well-characterized epilepsy genes were then investigated as potential targets of a pro-epileptogenic cellular signature in our simplistic cell culture model. Blockage of glutamatergic signaling by D,L-AP5 and NBQX and of the propagation of action potentials by TTX was performed to provide evidence for the principal role of neuronal excitation as trigger of the epigenetic machinery. This experimental strategy was designed to help answering the question if synchronized neuronal hyperexcitation is capable of inducing long-lasting epigenetic signatures and facilitating a cellular memory of epileptogenesis (CME).

## Materials and methods

### Animals and tissue preparation

Adult Wistar rats were obtained from Charles River (Sulzfeld, Germany), bred and maintained at the local animal center in breeding cages under controlled environmental conditions (12 h light/dark cycle, 20–23 °C, 50% relative humidity, drinking and feeding ad libitum). Newborn or up to two-day-old male and female offspring were used for the in vitro model. All animal experiments have been approved by the local animal care and use committee (TS-1/13) and were in accordance with the European Communities Council Directive and German Animal Welfare Act (54–2532.1-23/09, Directive 2010/63/EU).

### Preparation of cell suspensions and dispersed hippocampal cell culture

Cell suspensions were prepared from rat hippocampus as described previously [21]. Coverslips (for immunofluorescence staining and calcium imaging) and culture dishes (for any other application) were coated with poly-D-lysine hydrobromide (Sigma-Aldrich, Taufkirchen, Germany). Cell suspension was preplated onto an uncoated flask and incubated at 37 °C in 5% CO_2_ for 1 h. During this time glial cells settled down and adhered to the bottom of the flask, while neurons remained in the supernatant. Supernatant was collected thereafter and centrifuged at 800 rpm for 8 min at room temperature. Cell pellets were resuspended and cultured in serum-free Neurobasal-A medium supplemented with 2% B27, 0.5 mM GlutaMAX and 1% penicillin-streptomycin (all Life Technologies, Darmstadt, Germany). Cells were plated on poly-D-lysine coated dishes or coverslips at a density of 2.5 × 10^5^ onto 3.5 cm^2^. Cells were maintained at 37 °C in a fully humidified incubator containing 5% CO_2_. After 24 h Cytosine β-D-arabinofuranoside hydrochloride (AraC; Sigma-Aldrich) was added to inhibit proliferation of remaining glial cells. Neurons were maintained in dispersed culture with the original media up to 40 days in vitro (DIV).

### Glutamatergic excitation

Glutamate treatment of cell cultures was performed as described elsewhere [22, 23]. At 12 DIV*,* culture media was replaced by a physiological treatment solution (145 mM NaCl, 2.5 mM KCl, 10 mM HEPES [pH 7.4], including 10 mM glucose, 2 mM CaCl_2_, 1 mm MgCl_2_, and 2 μM glycine for control cultures, adding 10 μM glutamate for stimulation of the glutamate group). Neuronal cultures were exposed to treatment solution for 10 min, washed with treatment solution 3 times and replaced by original culture media again until the end of the experiment. 1 μM TTX (Sigma-Aldrich, Taufkirchen, Germany) was added during glutamate treatment to inhibit action potential discharges through interference with voltage-gated sodium channels. 10 μM 2,3-dihydroxy-6-nitro-7-sulfamoyl-benzo-quinoxaline-2,3-dione (NBQX, Signal-Aldrich) and 50 μM D-amino-5-phosphonovaleric acid (D,L-AP5, Sigma-Aldrich) were added to block excitatory synapses (i.e. AMPA/kainate and NMDA receptors) and prevent recurrent glutamatergic activity. After glutamate treatment neurons were kept in culture up to 4 weeks for downstream applications.

### Cell viability assay

Neuronal viability was determined 2 and 4 weeks after glutamate treatment with propidium iodide (PI) and fluorescein diacetate (FDA) (both Thermo Scientific, Dreieich, Germany). FDA diffuses through the membrane of viable cells where it is metabolized into the green fluorescent fluorescein. PI cannot pass the viable cell membrane but intercalates with DNA in necrotic cells. Neurons were incubated with 500 nM PI and 160 nM FDA for 5 min in Neurobasal A medium and washed 2 times with Neurobasal A medium. Semi-quantitative measurements of PI/FDA stained cells were performed in triplicate using a microcomputer imaging system (ColorView II CCD camera, Cell^F imaging software, Olympus, Tokyo, Japan) equipped to an Olympus XI70 microscope. PI and FDA positive cell bodies were tagged on the computer screen and manually counted in four independent visual fields at 20× objective magnification. Cell viability was determined three independent times in glutamate-treated cultures and time matched sham controls.

### Immunofluorescence staining

Immunofluorescence staining was performed 2 and 4 weeks after glutamate treatment. Neurons cultured on coverslips were fixed in 4% formaldehyde. Fixed cells were pre-treated with blocking solution containing tris-buffered saline (TBS; 0.05 M Tris-Cl pH 7.6) with 1% bovine serum albumin (BSA, Biochrom, Berlin, Germany), 2% fish skin gelatin (Sigma-Aldrich) and Triton X-100 (0.1%, Sigma-Aldrich, Steinheim, Germany) for 2 h. Primary antibodies were diluted in blocking solution and incubated overnight at 4 °C. GFAP (mouse monoclonal anti-GFAP, clone GA5, mAb3670, Cell Signaling, Danvers, USA) was used as glial marker at a dilution of 1:300 and MAP2C (chicken polyclonal anti-MAP2, ab5392, abcam, Cambridge, UK) as neuronal marker at a dilution of 1:2000. Secondary fluorophore-conjugated antibodies were diluted in blocking solution and incubated for 4 h at room temperature. Secondary antibodies were labelled with FITC (1:200, rabbit anti-chicken, ab6749, abcam) and Cy3 (1:100, goat anti-mouse, Dianova). Nuclei were counterstained with DAPI (Sigma-Aldrich) at a dilution of 1:1000 for 15 min. Semi-quantitative measurements of neuronal and glial cell numbers were performed in triplicate using a microcomputer imaging system (ColorView II CCD camera, Cell^F imaging software,) equipped to an Olympus XI70 inverted fluorescence microscope (Olympus, Tokyo, Japan). Immunohistochemically stained neuronal and glial cell bodies were tagged on the computer screen and manually counted at 20× objective magnification in four independent visual fields.

### Calcium imaging

Calcium imaging experiments were performed in triplicate during acute glutamate stimulation and two time points after glutamate treatment (3 d and 7 d). Coverslips were incubated with 5 μM Fluo-4 AM (Thermo Fisher) in recording solution (144 mM NaCl, 2.5 mM KCl, 2.5 mM CaCl_2_, 2.5 mM MgCl_2_, 10 mM Glucose, 10 mM HEPES, pH 7.4, 320 mOsm) for 30 min at 37 °C. After coverslips were washed twice with PBS, they were placed into a perfusion chamber filled with 500 μl recording solution. A Nikon TI-Eclipse inverted fluorescence microscope (Nikon, Tokio, Japan) with 10×, 0.45 NA objective and Perfect Focus System was used for all imaging experiments. Fluo-4 was excited by a Nikon Intensilight C-HGFI lamp (Neutral Density Filter 16) through excitation filters of 455–485 nm and a 495 nm dichroic long-pass mirror. The emitted light was passed through an emission bandpass filter of 500–545 nm (Semrock, Rochester, NY) and was projected onto a cooled EM-CCD camera (iXonEM DU-885, Andor). Imaging was controlled and recorded by NIS Elements (Nikon).

Images were recorded at 100 ms intervals and at an exposure time of 70 ms. Image stacks were converted into tagged image file format. All image analysis was performed using custom-written routines in MATLAB (The MathWorks, Natick, MA). Regions of interest (ROIs) were automatically detected using the background-determined feature point detection as described in Sbalzarini et al. [24] (intensity parameter w = 0.1–0.8%, approximate cell radius *r* = 5.0 pixel). For each ROI the relative fluorescence trace was calculated [25]. Spike estimation on these traces was performed defining a spike as an event of more than 3–6 standard deviations in amplitude within 30 time frames.

### Gene expression analysis

RNA was extracted from primary neuronal cultures at 5 different time points after glutamate treatment (3 h, 7 h, 24 h, 3 d, and 14 d) using TRIzol Reagent (Life technologies; according to the manufacturer’s instructions) followed by DNase treatment (Life technologies). First-strand cDNA synthesis was performed using the SuperScript II Reverse Transcriptase Kit (Life technologies; according to the manufacturer’s instructions). Quantitative real-time PCR was performed using the 7500 Fast Real-Time PCR System (Life technologies) with Power SYBR Green PCR Master Mix (Life technologies) as fluorescent dye according to the manufacturer’s protocol. Primers used for amplification of cDNA are specified in Table 1. GAPDH quantification was used as internal control for normalization. Fold difference of mRNA levels were calculated using the ΔΔCt method. No-template controls for each primer were included on every plate and melt curve analysis was performed to exclude unspecific amplification. All PCR reactions were performed in triplicate and repeated three independent times.

**Table 1:** cDNA primers used for quantitative real-time PCR for gene expression analysis

PrimerID	Forward primer	Reverse primer
Gria2	ACGAGTACATCGAGCAGAGGAA	GATGCCGTAGCCTTTGGAATC
Grin2a	TGGCCTCAGTGACAAGAAGTTC	AGACGGCTGCGTCATAGATGAA

### Chromatin immunoprecipitation assay

Chromatin immunoprecipitation (ChIP) was performed according to a modified X-ChIP protocol by abcam. Cultured neurons were cross-linked in 0.8% formaldehyde for 10 min at room temperature at 5 different time points after glutamate treatment (3 h, 7 h, 24 h, 3 d, 14 d). Crosslinking reaction was stopped by adding glycine to a final concentration of 0.125 M and centrifuged at 1000 rpm for 8 min at 4 °C. Pellet was washed twice in cold PBS and snap frozen in liquid nitrogen. Cells were lysed in SDS-lysis buffer (50 mM Tris-HCl [pH 8.0], 10 mM EDTA dihydrate, 1% SDS) for 1 h. Lysate was sheared using a Diagenode Bioruptor on high power for 4 × 5 cycles (30 s on-off). Equal amounts of chromatin lysate for glutamate and control group (1500–3000 ng) were diluted ten times with RIPA Buffer (50 mM Tris-HCl [pH 8:0], 150 mM NaCl, 2 mM EDTA dihydrate [pH 8.0], 1% NP-40, 0.5% Sodium deoxycholate, 0.1% SDS) and pre-cleared with Dynabeads Protein G magnetic beads (Life technologies) for 1 h. Immunoprecipitation was performed overnight with the corresponding primary IgG antibody along with the Dynabeads Protein G magnetic beads. Immune complexes were washed three times with ChIP wash buffer (0.1% SDS, 1% Triton X-100, 2 mM EDTA dihydrate [pH 8.0], 150 mM NaCl, 20 mM Tris-HCl [pH 8.0]), once with ChIP final wash buffer (0.1% SDS, 1% Triton X-100, 2 mM EDTA dihydrate [pH 8.0], 500 mM NaCl, 20 mM Tris-HCl [pH 8.0]) and were then eluted from the beads with 120 μl elution buffer (1% SDS, 0,1 M NaHCO_3_, pH 9.0). Protein-DNA cross-links were reverted by overnight incubation at 65 °C and DNA was extracted using the QIAquick PCR Purification Kit (Qiagen) according to the manufacturer’s protocol. Immunoprecipitated DNA was preamplified using the SsoAdvanced PreAmp Supermix (Biorad, Ismaning, Germany) according to the manufacturer’s instructions. DNA quantification was done by quantitative real-time PCR using the same protocol as mentioned above. The following antibodies were used: 2 μg H3K4me3 (07–473, Millipore, Molsheim, France), 2 μg H4ac (06–866, Millipore), 4 μg H3K27me3 (07–449, Millipore), 6 μg H3K9me3 (ab8898, abcam) and IgG (Millipore). Primers used for preamplification and qPCR of immunoprecipitated DNA are shown in Table 2. Hist1H4B was used as control primer for normalization for H4ac, H3K4me3, MyoD for H3K27me3, and Zfp12 for H3K9me3. All steps were performed at 4 °C up to and including the immunoprecipitation step. PBS, SDS-lysis buffer and RIPA buffer were supplemented with protease inhibitors (EDTA-free protease inhibitor cocktail tablets; Roche) and deacetylase inhibitors (Active Motif, La Hulpe, Belgium) immediately before application. Non-specific IgG antibody was included as a negative control to exclude unspecific interaction. All ChIP experiments were repeated three independent times for each antibody.

**Table 2:** Genomic DNA primers used for preamplification and quantitative real-time PCR to amplify immunoprecipitated DNA

PrimerID	Forward primer	Reverse primer
Gria2 A	TGGGAGTCGTCCTTTCAGAGA	AAACTGATTTCCGGTTGCTATG
Gria2 B	CGCTGTCCTCGGTGCTAAAAT	AGAGAGGGGCAGGCAGTCT
Gria2 C	TGTGCGCGCTCGTGTGAGA	TCCTTATTTCCCAGTTGTAGCT
Grin2a A	AGCCAGGGCTCTAGAAGAGA	GCGACGAGCCGGGAGAAGA
Grin2a B	GTGGAGGTTCCCACTAAGCTT	AGGGCGGAGGAGAGATGGA

### Bisulfite sequencing

Genomic DNA was extracted from primary neuronal culture 4 weeks after glutamate treatment using the QIAamp DNA Micro Kit (Qiagen, Hilden, Germany), followed by bisulfite conversion of 1 μg of genomic DNA using the EpiTect Bisulfite Kit (Qiagen) according to the manufacturer’s protocol. Regions of interest were amplified using the TaKaRa EpiTaq HS Kit (TaKaRa Clontech, Otsu, Japan) and cloned using the TOPO TA Cloning Kit (Life technologies) doing blue/white screening. White colonies were selected and grown in LB medium overnight. The plasmid was purified using the GeneJET Plasmid Miniprep Kit (Thermo Scientific, Darmstadt, Germany) and clones were sequenced by Sanger sequencing (GATC Biotech, Cologne, Germany). Sequences were quality controlled and aligned using the CLC sequence viewer (CLC bio) and Quantitation tool for Methylation Analysis software (RIKEN). Primers used to amplify bisulfite converted DNA are summarized in Table 3.

**Table 3:** Bisulfite primers used for DNA methylation analysis

PrimerID	Forward primer	Reverse primer
Gria2 BB1	TTAGTTGGGTTAGGTGAGGTA	TCCCATACACTCACACAATCA
Gria2 BB2	TATAGAGAGATAAAGATAGAGAGAT	AATAAATTACCTCATTACATCAAAC
Grin2a BB1	AGGTATTGAGAGGAGTATTTTGG	CCACCACACCAACTTAAAACT
Grin2a BB2	AGTGAGTGATAAAAGTAGTTAGTG	ATCTCTTCCTAACCTTACCTTAT

### Statistical analysis

Statistical analysis was performed using GraphPad Prism6 (GraphPad Software, San Diego, CA). Graphs were generated using MATLAB (The MathWorks Inc., Natick) or GraphPad. Error bars and specified values represent standard deviation (SD). Depending on number of groups analyzed, sample size and respective distribution, statistical comparisons were made with Fisher’s exact test (site-specific DNA methylation), one-way ANOVA and Dunnett’s correction for multiple comparisons (gene expression, calcium imaging and ChIP data), or Mann-Whitney test (IF/cell counts, DNA methylation), respectively. In all figures, asterisks indicate a level of significance of *p* < 0.05.

## Results

**Fig. 1:**
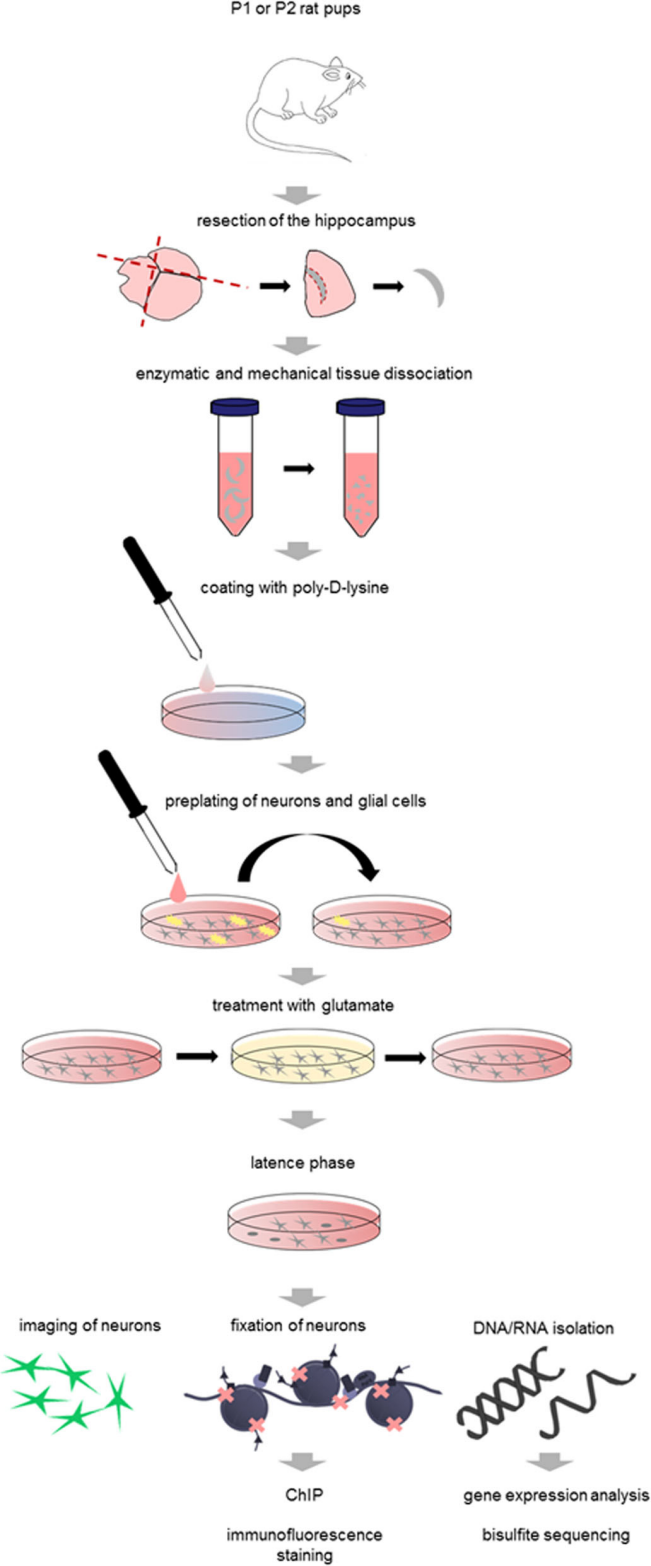
Experimental study design. Brains of P0 to P2 rat pups were removed, hippocampi resected and then enzymatically and mechanically dissociated. Culture dishes were coated with poly-D-lysine. Cell suspension was pre-plated for 1 h, to separate glial (*yellow*) and neuronal cell populations (*grey*) by sedimentation. Neuronal cell population was cultured on poly-D-lysine coated dishes. After 12 DIV neurons were treated with 10 μM glutamate for 10 min. Cells were further cultured for up to 4 weeks after glutamate injury and subsequently imaged, fixed with formalin or lysed for downstream applications

Transient exposure to glutamate has been shown to induce spontaneous ictal-like activity in hippocampal neuronal cultures. In the present study we used this simplistic neuronal cell culture model to analyze how massive excitation of neuronal membranes modifies the epigenetic machinery and translates into persisting gene expression changes. Rat hippocampal neurons were cultured 12 DIV and then exposed to 10 μM glutamate for 10 min. Cultures were kept up to 4 weeks under standard conditions for further use of specific downstream applications (see below and summary in Fig. 1).

### Viability of dissociated rat hippocampal neurons

**Fig. 2:**
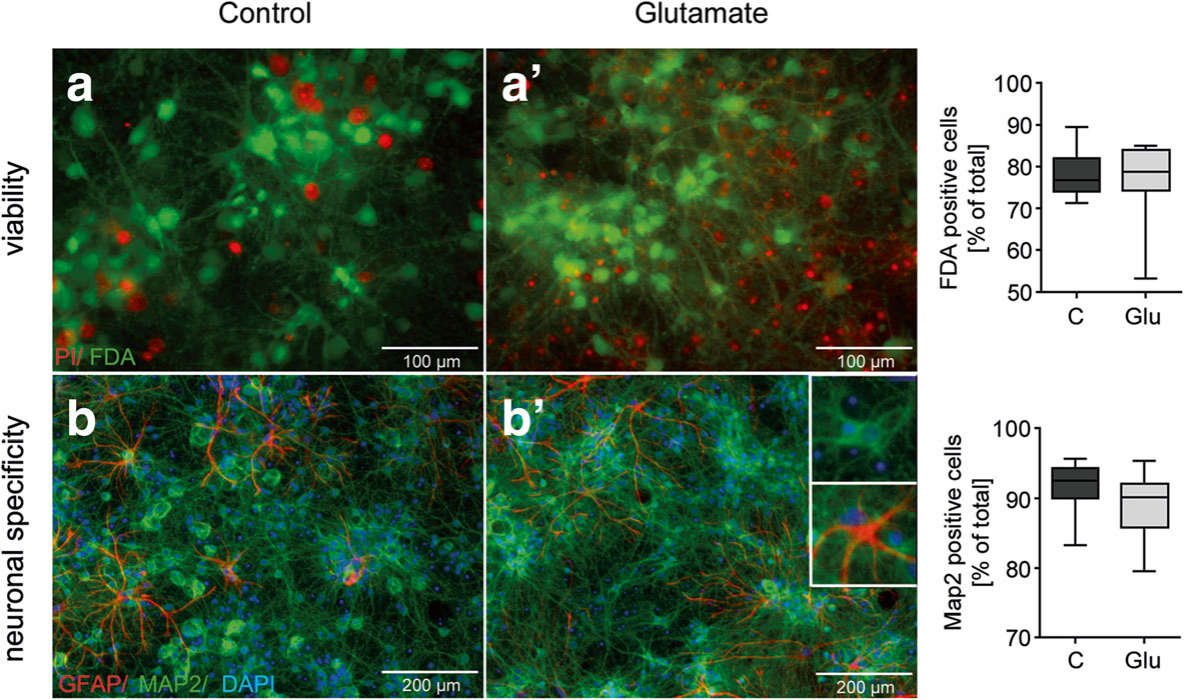
Effect of glutamate treatment on cell viability, morphology and specificity using bright field and fluorescence microscopy. **a** Live/dead double-staining of neuronal culture based on differential uptake of PI and FDA 4 weeks after treatment. Vital cells stained green whereas dead cells stained red. No difference was seen between the number of surviving cells following glutamate treatment compared to time-matched sham controls (*p* > 0.05). **b** Cell morphology of neurons 4 weeks after glutamate injury was imaged by bright field light microscopy. Double-immunofluorescent staining of cultured cells with neuronal marker MAP2 (*green*) and glial marker GFAP (*red*) 4 weeks after glutamate treatment is shown. Nuclei were counterstained using DAPI (*blue*). No difference was seen between the relative number of neuronal cells in our preparations following glutamate treatment compared to time-matched sham controls (*p* > 0.05)

Using PI/FDA stainings for assaying cell viability, 77% (+/−1%) of cells survived 4 weeks after transient 10 μM glutamate exposure, compared to 78% (+/− 2%) in time-matched sham-treated controls (Mann-Whitney test, *p* > 0.05; Fig. 2a and a’). These cells established a complex neuronal network. Double immunofluorescence with the neuronal marker MAP2C and glial marker GFAP identified 89% (+/− 1%) of neurons in the cultured cell population compared to 92% (+/− 1%) in time-matched sham-treated controls 4 weeks after glutamatergic stimulation (Mann-Whitney test, *p* > 0.05; Fig. 2b and b’).

### Glutamate induced alterations in neuronal excitability, development of epileptogenic networks

**Fig. 3:**
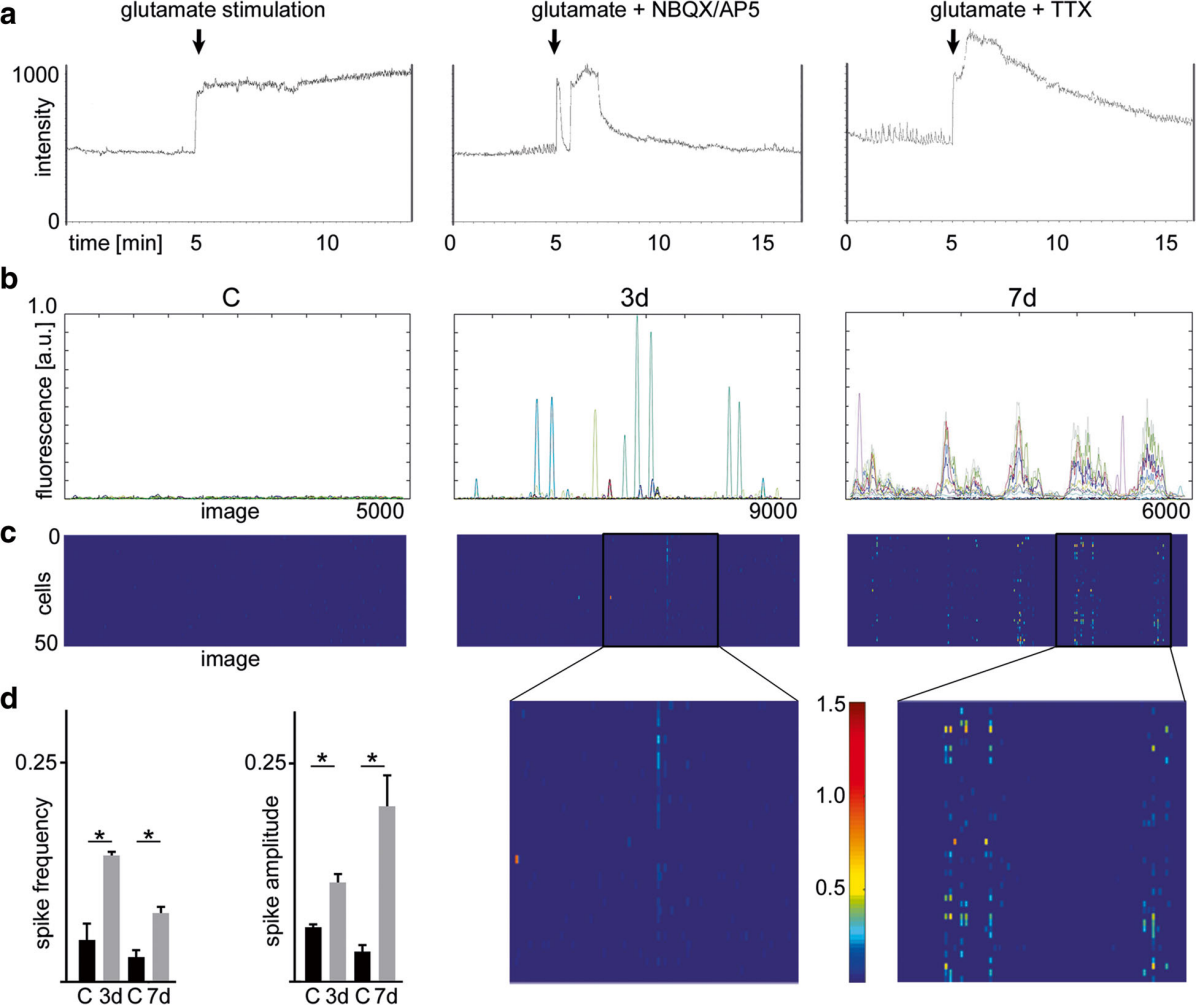
Development of spontaneous recurrent epileptiform discharges following glutamate injury in cultured rat hippocampal neurons. Calcium imaging. **a** Development of neuronal activity measured by calcium uptake and release of a single selected neuron before, during and after stimulation with glutamate and glutamate supplemented with either NBQX/AP5 or TTX. **b** Display of the development of synchronized neuronal activity determined by calcium imaging of 10 representative neurons 3 and 7 days after glutamate injury compared to sham control. **c** Heatmap showing exemplarily the intensity and frequency of calcium signals of 50 simultaneously recorded neurons 3 or 7 days after stimulation with glutamate. Insets highlight synchronization and bursting activity. **d** Mean of spike frequency and spike amplitude of recorded neurons 3 and 7 after glutamate injury is significantly increased compared to corresponding controls. All error bars represent standard deviation. Asterisks indicate significance (*p* < 0.05). AP5 - D-amino-5-phosphonovaleric acid; C – control; d – days; NBQX - 2,3-dihydroxy-6-nitro-7-sulfamoyl-benzo-quinoxaline-2,3-dione; TTX – tetrodotoxin

Next we asked if glutamatergic excitation changed the intrinsic network activity of cultured hippocampal neurons. Using live cell calcium (Ca^2+^) imaging with Fluo4-AM dye, we monitored glutamate-evoked recurrent spontaneous Ca^2+^ transients as surrogate marker for spiking activity in neuronal networks 3d and 7d after glutamate stimulation. Twenty minute traces of spontaneous Ca^2+^ activity, were simultaneously captured from neuronal populations of up to 200 cells at single cell resolution. Isolated episodes of low frequency and low amplitude Ca^2+^ oscillations could be observed in dissociated hippocampal neurons. Following glutamate exposure a significant increase in intracellular Ca^2+^ uptake was identified (Fig. 3a). This could be reverted to baseline, but not completely blocked, by either NBQX/AP-5 or TTX application (Fig. 3a and b). Over time we detected a significant increase in amplitude and frequency of spikes (from 3d on), synchronization of neuronal firing over large networks of neurons as well as a shift from single spikes towards bursts of spikes following glutamate stimulation (from 7d on) compared to sham-treated time-matched controls (Fig. 3c). Cells that were co-treated with either NBQX/AP5 or TTX and glutamate showed little synchronization and no burst activity similar to sham controls (data not shown). Taken together, our data is compatible with a simplistic pathogenic model of epileptogenesis.

### Glutamate receptor subunit expression in epileptogenesis

**Fig. 4:**
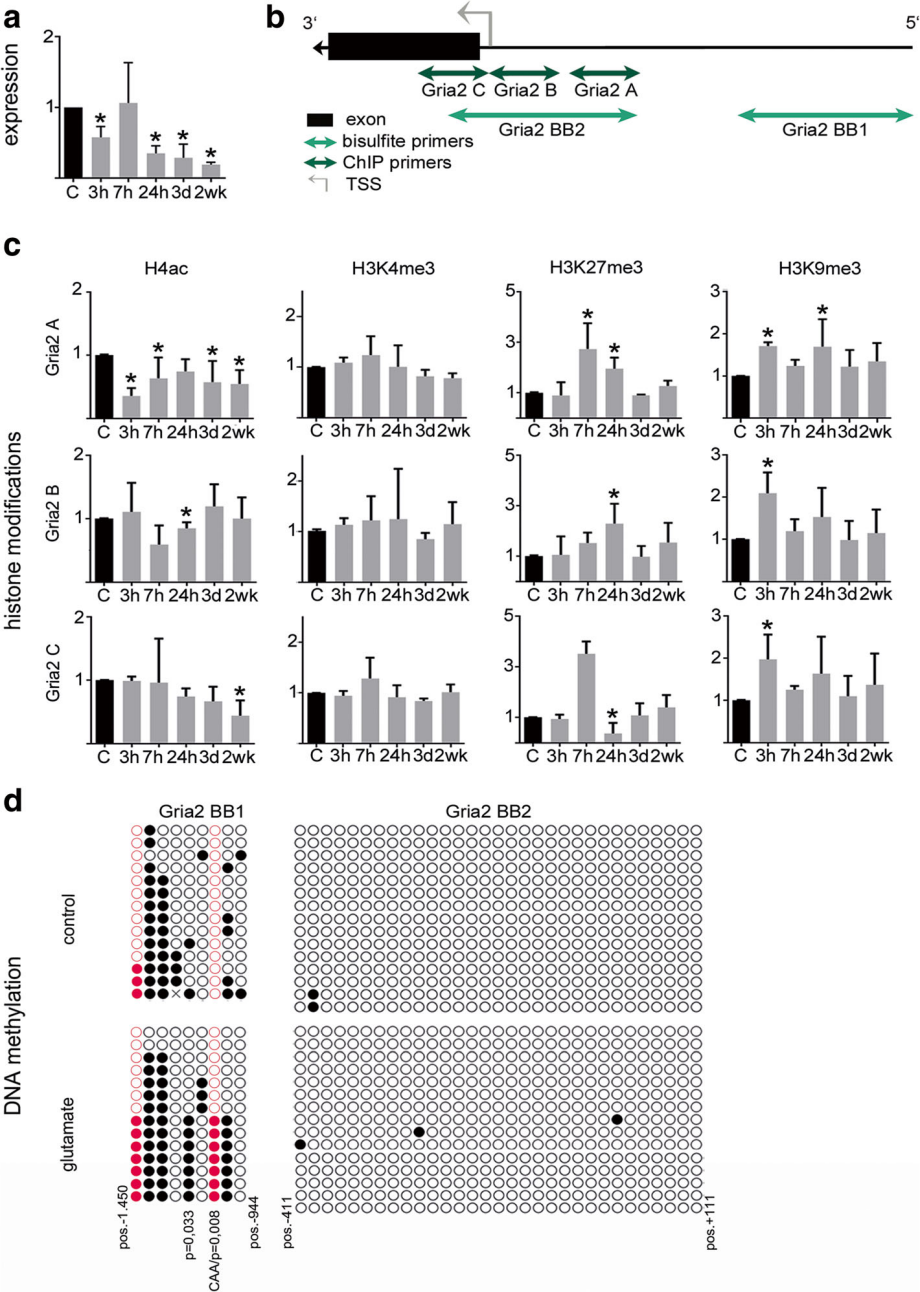
Decreased Gria2 gene expression correlates with dynamic regulation of Gria2 gene promoter histone modifications. **a** Relative quantification (2^-∆∆Ct^) of Gria2 mRNA levels at 5 different time points (3 h, 7 h, 24 h, 3 d and 2 weeks) after glutamate treatment compared to control treatment. **b** Schematic presentation of Gria2 gene promoter region and amplicon localization for qPCR of immunoprecipitated DNA and bisulfite PCR used for DNA methylation analysis. **c** Chromatin immunoprecipitation of histone modifications H4ac, H3K4me3, H3K27me3 and H3K9me3 at the promoter region of Gria2 at 5 different time points (3 h, 7 h, 24 h, 3 d; 2 weeks) after transient glutamate stimulation. Data are expressed as mean fold change over control treatment plus standard deviation after normalization to positive control region of the corresponding antibody. **d** Bisulfite sequencing of the Gria2 promoter identified increased DNA methylation of glutamate-treated neuronal cultures compared to sham controls at single CpG (*black dots*) and non-CpG (*red dots)* positions. Positions of analyzed loci relative to TSS, non-CpG sequences and *p*-values of Fisher’s exact test of significant locus specific differences in methylation are shown. All error bars represent standard deviation. Asterisks indicate significance (*p* < 0.05)

**Fig. 5:**
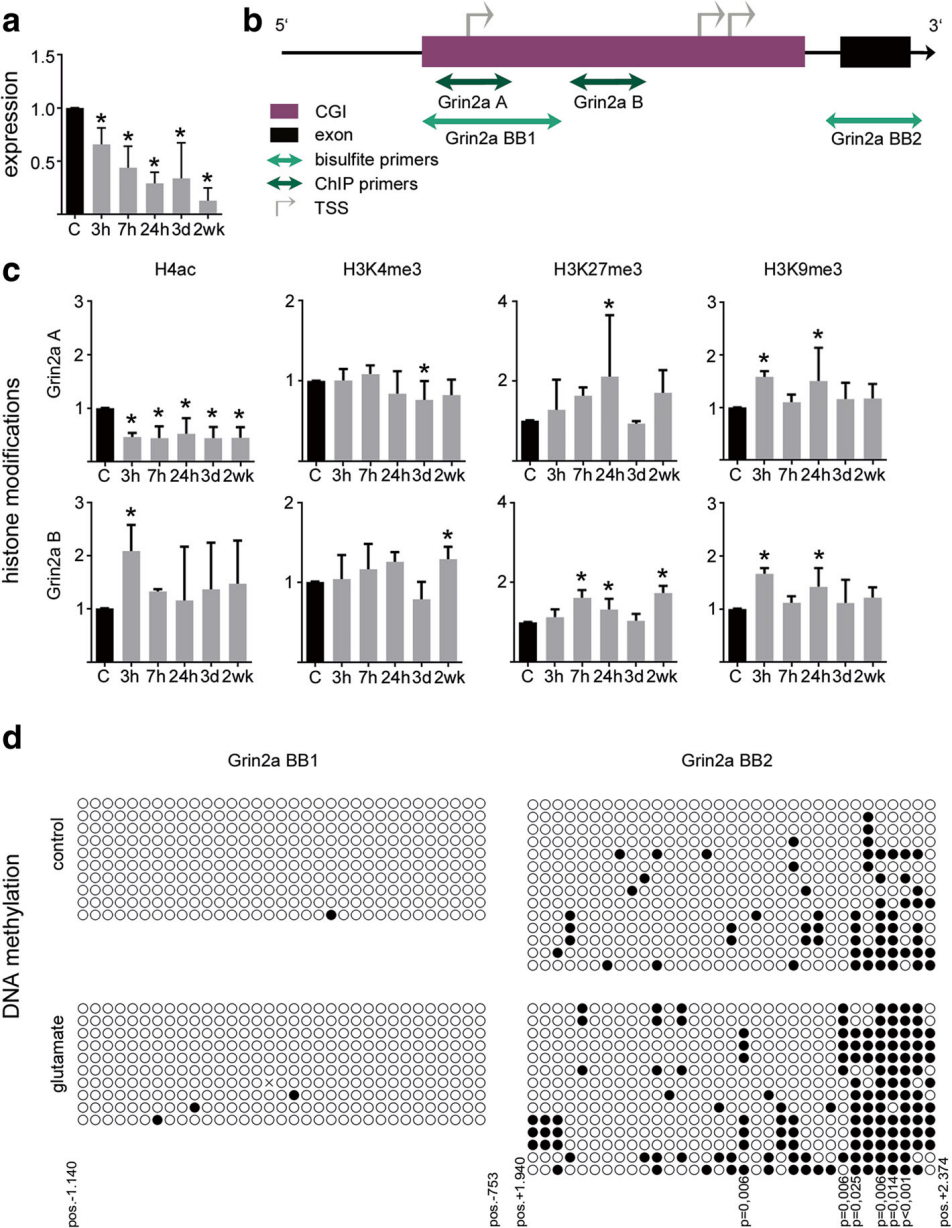
Decreased Grin2a gene expression correlates with dynamic regulation of Grin2a gene promoter histone modifications. **a** Relative quantification (2^-∆∆Ct^) of Grin2a mRNA levels at 5 different time points (3 h, 7 h, 24 h, 3 days and 2 weeks) after glutamate treatment compared to time-matched sham controls. **b** Schematic presentation of Grin2a gene promoter region and amplicon localization for qPCR of immunoprecipitated DNA and bisulfite PCR used for DNA methylation analysis. **c** Chromatin immunoprecipitation of histone modifications H4ac, H3K4me3, H3K27me3 and H3K9me3 at the promoter region of Grin2a at 5 different time points (3; 7; 24 h, 3 days; 2 weeks) after transient glutamate stimulation. Data are expressed as mean fold change over time-matched sham controls after normalization to positive control region of the corresponding antibody. **d** Bisulfite sequencing of the Grin2a promoter identified increased DNA methylation in glutamate-treated neuronal cultures compared to time-matched sham controls. Positions of analyzed loci relative to the most downstream TSS and p-values of Fisher’s exact test of significant locus specific differences in methylation are show. All error bars represent standard deviation. Asterisks indicate significance (*p* < 0.05)

Altered expression or functional mutations affecting glutamate receptor subunit composition have been previously linked with epilepsy. We studied epigenetic gene expression of ionotropic α-amino-3-hydroxy-5-methyl-4-isoxazolepropionic acid (AMPA) receptor subunit *Gria2* and N-methyl-D-aspartate (NMDA) receptor subunit *Grin2a* at 5 different time points after glutamate stimulation (i.e. 3 h, 7 h, 24 h, 3 d, and 2 w). Using qPCR analyses, we identified a consistent reduction in *Gria2* and *Grin2a* gene expression at all experimental time points. *Gria2* mRNA levels were significantly decreased after 3 h and declined further until 2 weeks after glutamate treatment (Fig. 4a; one-way ANOVA and Dunnett’s post-hoc test *p* < 0.0001). Likewise, *Grin2a* mRNA levels decreased consecutively from 3 h to 2 weeks after glutamate stimulation (Fig. 5a; *p* < 0.0001). These indings support previous data describing similar changes in subunit composition of glutamate receptors in experimental and human epilepsy (Table 4).

**Table 4:** Epilepsy-associated genes previously reported to be epigenetically regulated

Epilepsy-ass. Gene	Previously described epigenetic modification	Organism and Experimental condition	Reference
*Bdnf*	DNA methylation	Rat HC primary neuronal culture (high K+)	[11]
	H3/H4 acteylation	Rat ECS model	[18]
	DNA methylation	Rat SE model (KA)	[13]
	Chromatin remodeling	Rat SE model (KA)	[48]
*c-Fos*	H3 phosphorylation	Mouse SE model (KA)	[49]
	H3/H4 acetylation	Rat ECS model	[18]
	H4 acetylation	Mouse SE model (KA)	[17]
*Cpa6*	DNA methylation	Human TLE-HS	[8]
*Creb*	H3/H4 acetylation	Rat ECS model	[18]
*Gria2*	H4 acetylation	Rat SE model (PILO)	[15]
*Grin2a*	H4K12 acetylation	Animal models of AD	[30]
	DNA methylation	Human Depression	[31]
*Grin2b*	DNA methylation	Rat SE model (KA)	[13]
*Reelin*	DNA methylation	Human TLE-HS	[10]
Genome-wide alterations	DNA methylation	Rat SE model (PILO)	[50]
		Rat SE model (KA)	[51]
		Rat SE model (KA)	[14]
		Mouse SE model (KA)	[51]
		Human TLE-HS	[12]
		Rat SE model (SSSE), Rat TBI model, and Rat SE model (PILO)	[9]

### Epigenetic control of glutamatergic mechanisms in epileptogenesis

We then asked whether altered *Gria2* and *Grin2a* expression can be linked to suppressive epigenetic histone modifications [15, 16, 26]. We observed a significant and permanent decrease in H4ac levels within the *Gria2* promoter up to 2 weeks post injury, both up- and downstream the transcriptional start site (TSS) including the 5′ untranslated region (5’UTR) and part of exon 1 of the *Gria2* gene (Fig. 4b and c, left panel, locus Gria2 A, one-way ANOVA and Dunnett’s post-hoc test *p* < 0.0001; locus Gria2 C, *p* < 0.01). NMDA receptor subunit *Grin2a* also showed permanent decrease in promoter associated acetylation levels of histone H4 at a region corresponding to the most upstream of three alternative TSSs (Fig. 5b and c, left panel, locus Grin2a A, *p* < 0.0001).

No changes in H3K4 trimethylation (H3K4me3; *p* > 0.05), another gene activating histone modification, were found at any given locus of the *Gria2* and *Grin2a* promoter following glutamate injury compared to sham controls (Figs. 4c and 5c, second panel on left, *p* > 0.05).

In contrast, repressive H3K9 trimethylation (H3K9me3) increased in a time-dependent manner peaking at 3 h and 24 h after glutamate treatment providing an inhibitory signal at the promoter structures of *Gria2* and *Grin2a* (Figs. 4c and 5c, right panel, *p* < 0.05). H3K27me3 also increased significantly at *Gria2* and *Grin2a* promoters (Fig. 4c and 5c, second panel on right, *p* < 0.01), thereby adding, with a slight delay over H3K9me3, another repressive signal at these gene structures. Both signals were transient and returned to control levels at 3 d after glutamate exposure. As long-term gene silencing may be mediated through DNA methylation we performed bisulfite cloning and sequencing of the *Gria2* and *Grin2a* promoters including TSSs, 5′ UTRs and parts of exon 1 at 4 weeks following glutamate stimulation and in time-matched sham-treated controls. We identified increased DNA methylation at both CpG and non-CpG sites within promoter regions of both *Gria2* (Fig. 4d) and *Grin2a* (Fig. 5d).

Taken together our data indicated that glutamate induced massive neuronal excitation induced changes in histone modifications and DNA methylation at the promoter regions of ionotropic glutamate receptor subunits compatible with a collaborative epigenetic regulation of gene expression.

### Inhibition of excitatory glutamatergic signaling rescues epigenetic modifications of epilepsy candidate genes

**Fig. 6:**
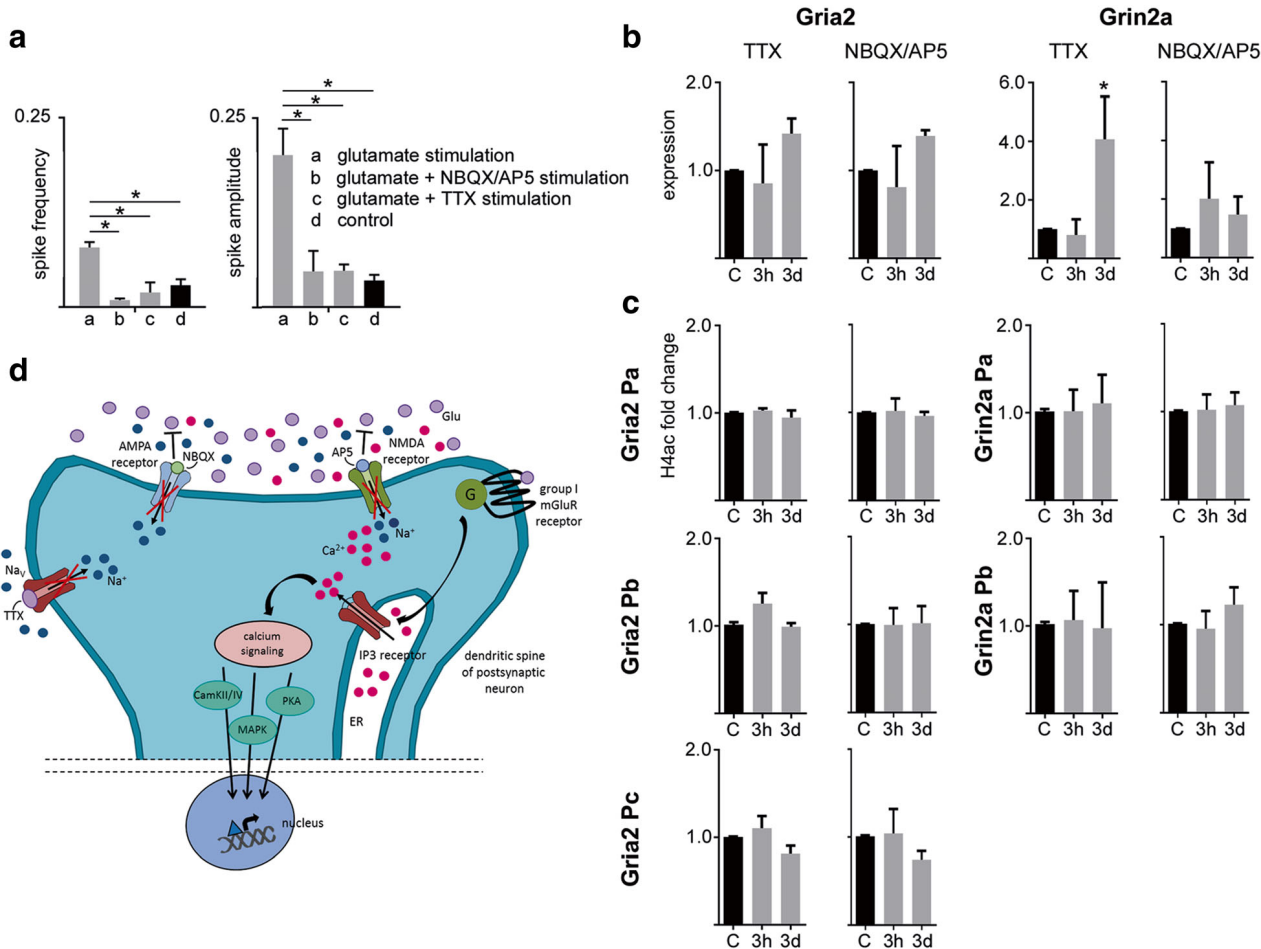
Inhibition of glutamatergic signaling partially rescues aberrant gene expression of epilepsy candidate genes and associated epigenetic changes. **a** Calcium imaging 7 days after stimulation with glutamate, glutamate and NBQX/AP5, glutamate and TTX or sham controls. Mean of spike frequency and spike amplitude of recorded neurons during the recorded period of time are shown. **b** Relative quantification (2^-∆∆Ct^) of Gria2 and Grin2a mRNA levels at 2 different time points (3 h and 3 days) after glutamate exposure with TTX or NBQX/AP5 compared to control treatment. **c** Chromatin immunoprecipitation of histone modification H4ac at the promoter region of Gria2 and Grin2a at 2 different time points (3 h and 3 days) after glutamate treatment with TTX or NBQX/AP5. Data are expressed as mean fold change over control treatment plus standard deviation after normalization to positive control region of the corresponding antibody. **d** Schematic presentation of the effect of inhibitors TTX, NBQX and AP5 and glutamate signaling as well as common calcium signaling pathways translating external cues into changes in gene expression. AMPA - α-amino-3-hydroxy-5-methyl-4-isoxazolepropionic acid; AP5 - D-amino-5-phosphonovaleric acid; C – control; CaMK – Calcium/Calmodulin dependent kinase; d – days; Glu – glutamate; h – hours; MAPK – MAP kinase; mGluR – metabotropic glutamate receptor; NBQX - 2,3-dihydroxy-6-nitro-7-sulfamoyl-benzo-quinoxaline-2,3-dione; NMDA - N-methyl-D-aspartate; PKA – protein kinase A; TTX – tetrodotoxin; w - weeks

To test whether epigenetic alterations were induced by neuronal hyperactivity in our cell culture we performed inhibition studies either interfering with excitatory synapses or propagation of action potentials (Fig. 6). Neuronal cultures were simultaneously treated with glutamate and NBQX/AP5 or TTX, respectively. Calcium imaging performed 7 d after treatment showed little synchronization, no burst activity (data not shown) and significantly decreased spike frequency and amplitude, similar to sham controls (Fig. 6a). Cell viability assays and quantification of neuronal and glial cells in culture identified cell survival rates similar to time-matched sham-treated control cultures two weeks following treatment (data not shown). TTX and NBQX/AP5 prevented glutamate induced gene repression of *Gria2* and *Grin2a* (Fig. 6b). As an indicator of epigenetic changes H4ac levels were determined 3 h and 3 d after treatment with TTX or NBQX/AP5. Glutamate induced decrease of H4ac at *Gria2* and *Grin2a* could be abolished by TTX and by NBQX/AP5 (Fig. 6c, with no difference to sham-treated controls. Taken together, the results demonstrated a rescue of aberrant glutamate receptor subunit gene expression and regulatory epigenetic marks after treatment with TTX or NBQX/AP5.

## Discussion

Understanding the sequence and timing of molecular epileptogenesis is mandatory for targeted therapies and disease-modifying intervention. The hippocampus is key to ictogenesis in TLE and likely encodes structural, molecular and functional signaling pathways promoting chronic epilepsy. We asked whether and how massive hyperexcitation of neurons translates into aberrant and persisting pro-epileptogenic gene expression and upstream epigenetic modifications, thereby contributing to a cellular memory of epileptogenesis (CME). The divergent cellular composition of CNS tissue with glia, neurons and mesenchyme makes it challenging to unravel such complex issue [27]. A “simplistic” neuronal cell culture model of rat hippocampal neurons was specifically used to address this question.

Following transient glutamatergic stimulation [23] we studied cellular and molecular changes in principal hippocampal neurons at different time points up to 4 weeks after glutamatergic stimulation. We recorded rhythmic neuronal activation at 7 days after glutamatergic stimulation, and identified complex epigenetic alterations leading to decreased expression of excitatory glutamate receptor genes *Gria2* and *Grin2a*. Inhibition of ionotropic glutamatergic signaling and propagation of action potentials with NBQX/AP5 and TTX, respectively, during glutamate stimulation rescued aberrant gene expression and epigenetic modifications in cultured neurons. Moreover, the time-dependent development of epileptiform neuronal activation was blocked by this treatment. *Gria2* and *Grin2a* are well recognized candidate genes of epileptogenesis [28, 29]. Previous studies identified transcriptional regulation of *Gria2* by epigenetic mechanisms (Table 4; [15]). *Grin2a* expression was previously linked to HDAC2 activity and H4K12acetylation in animal models of Alzheimer’s disease [30]. Moreover, aberrant DNA methylation at the *GRIN2A* locus was described in patients with major depression [31]. Our model revealed fast decrease in both *Gria2* and *Grin2a* gene expression following glutamate induced neuronal hyperactivity. Downregulation of glutamate receptor subunits was initiated within 3 h following glutamate exposure and remained stable thereafter. No downregulation of *Gria2* and *Grin2a* was observed upon inhibition of glutamatergic excitation or propagation of action potentials with NBQX/AP5 or TTX, respectively. Our data were in line with previous findings showing that glutamate receptor subunit composition can be adjusted to neuronal activity within minutes or hours [32]. Long-term downregulation of *Gria2* and *Grin2a* are suggested to contribute to lasting changes in AMPA and NMDA receptor properties and downstream pro-epileptogenic events including neuronal death and functional network reorganization [33–35]. Changes in synaptic strength all over the network may thus have formed the basis for the development of rhythmic neuronal network hyperactivity as shown in our model and monitored by calcium imaging. Future studies are required to gain further insight into signaling over and synchronization of the neuronal network in the present model. Temporal resolution of our imaging experiment was too low to reliably dissect spike timing and temporal recruitment of cells into the active neuronal network. Moreover extended imaging of glutamate-treated cultures at earlier (e.g. 24 h and 48 h) and later time-points (e.g. 14 d and 4 weeks) will be needed to understand the physiological properties of the cells in relation to observed molecular changes.

In our particular interest to understand regulatory events of altered gene expression in epileptogenesis, we observed long-lasting decrease in histone 4 acethylation (H4ac) at *Gria2* and *Grin2a* promoters. Histone acetylation promotes a chromatin structure permissive to gene expression [36]. There are 26 sites of acetylation on a nucleosome and histone acetylation is dynamically regulated by histone acetyltransferases (HATs) and the antagonistic effects of histone deacetylases (HDACs) to adjust the level of transcription. Both *Gria2* and *Grin2a* were previously shown to be sensitive to H4 deacetylation by HDACs [30].

Beside histone deacetylation we identified a rapid but transient increase in inhibitory H3K9me3 over the first 3 h following the glutamatergic excitation. Although H3K9me3 is mostly recognized for its role in stabilizing heterochromatin [37], recent studies provided evidence that H3K9me3 contributes also to short-term silencing of actively transcribed genes [38, 39]. There was also a delayed and transient increase in H3K27me3, another transcriptional repressing histone modification, occurring 7 h hours to one day after glutamate stimulation. Fast but transient changes in histone methylation indicate that they mediate transcriptional repression, but are not used for long-term gene silencing. Therefore, we analyzed DNA methylation patterns 4 weeks after glutamate stimulation. DNA methylation is commonly established at a much slower rate than histone modifications and rather contribute to stabilizing silencing marks than to initiate transcriptional repression [40]. Machnes et al. previously showed that kainic acid exposure to mouse hippocampal slices as well as kainic acid induced status epilepticus in rats caused a mild increase in DNA methylation at the 5′ regulatory region of the *Gria2* gene [26]. Promoter methylation correlated with suppression of *Gria2* mRNA expression. Also, in patients with major depressive disorder altered DNA methylation had been reported for three distinct CpG sites along the *GRIN2A* promoter [31]. Here we identified increased CpG and non-CpG methylation in the *Gria2* promoter after glutamate injury and seizure-like events in our model suggesting a role in long-term down-regulation of the gene.

### Time dependent epigenetic patterns may correlate with disease progression

**Fig. 7:**
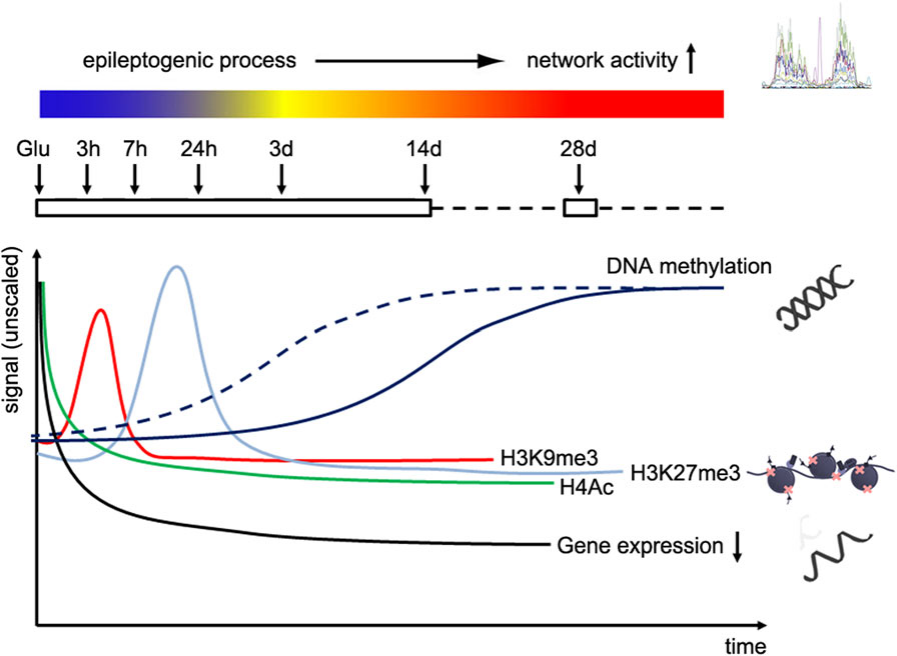
Molecular pathogenic model of gene repression in epileptogenesis. The present in-vitro model some captures electrophysiological and molecular features of epileptogenesis on a compressed scale. The latent period after glutamate (Glu) injury was characterized by silencing of epilepsy candidate genes (*black line*) through convergent action of different epigenetic mechanisms including locus-specific loss of histone acetylation (green), transient increase in H3K9 (*red*) and H3K27 trimethylation (*light blue*), and accumulation of DNA methylation at respective gene promoters (*dark blue*; dotted and solid lines indicate different possible pathogenic time courses for DNA methylation accumulation), associated gradual onset of neuronal spiking activity after 3 days and later synchronization of neuronal hyperactivity as well as a change in signal towards bursting activity after 7 days

The present study allowed us to analyze long-term dynamics of epigenetic mechanisms from the time point of glutamate-induced massive neuronal hyperactivity through a latency period until the onset and presumed maintenance of recurrent hypersynchronous activity (Fig. 7). We detected both, transient gene specific and long-lasting epigenetic modifications. Fast changes detected in histone acetylation corresponded well to early changes in gene expression at both gene promoters. This is in line with other studies showing that histone acetylation turnover mediates fast responses to environmental signals. We determined fast and transient H3K9me3 at the promoter and first exon of *Gria2* and *Grin2a* accompanying transcriptional repression, which suggested that H3K9me3 is involved in the initiation of transcriptional repression, but not maintenance. Alterations in H3K27me3 emerged later within 1 day after glutamate stimulation and were transient. These findings also confirm previous hypotheses that histone modifications are not stable enough to mediate long-term transcriptional changes. DNA methylation patterns are in general regarded less dynamic than histone modifications, but long-lasting. This could be confirmed in our cell culture model by increased CpG methylation at both the *Grin2a* and *Gria2* promoters.

## Conclusion

Our data linked the pathogenic model of gene repression in epileptogenesis with the regulatory epigenetic machinery of the cell. Persisting downregulation of both target genes was initiated through loss of H4 acetylation, accompanied by transient increase in H3K9me3, and subsequently stabilized by establishing H3K27me3. Finally, gene-specific repression is locked by DNA promoter methylation, leading to a cellular memory of epileptogenesis (Fig. 7). Our model may be further used to decipher the signaling pathway from an excited neuronal membrane to the cell nucleus, addressing key regulatory events of the epigenetic machinery for other epilepsy target genes of interest, asking for communalities as shared pathomechanism of epileptogenesis, and also looking into epigenetic mechanisms of previously described gene activation (e.g. RE1 Silencing Transcription Factor/Rest [41], neurotransmitter receptors mGluR1 and 4 [42], or Ca2+ channels [43]). Different mechanisms including Ca^2+^ signaling (Fig. 6e; [44, 45]) and metabolic regulation of transcription factors have been already proposed [46, 47] and may be the missing link between neuronal hyperactivity, epigenetic gene regulation and CME.
